# mTOR: A Cellular Regulator Interface in Health and Disease

**DOI:** 10.3390/cells8010018

**Published:** 2019-01-02

**Authors:** Fahd Boutouja, Christian M. Stiehm, Harald W. Platta

**Affiliations:** Biochemie Intrazellulärer Transportprozesse, Ruhr-Universität Bochum, 44801 Bochum, Germany; fahd.boutouja@rub.de (F.B.); Christian.stiehm@rub.de (C.M.S.)

**Keywords:** mTOR, autophagy, kinase, phosphorylation, aging, cancer

## Abstract

The mechanistic target of Rapamycin (mTOR) is a ubiquitously-conserved serine/threonine kinase, which has a central function in integrating growth signals and orchestrating their physiologic effects on cellular level. mTOR is the core component of differently composed signaling complexes that differ in protein composition and molecular targets. Newly identified classes of mTOR inhibitors are being developed to block autoimmune diseases and transplant rejections but also to treat obesity, diabetes, and different types of cancer. Therefore, the selective and context-dependent inhibition of mTOR activity itself might come into the focus as molecular target to prevent severe diseases and possibly to extend life span. This review provides a general introduction to the molecular composition and physiologic function of mTOR complexes as part of the Special Issue “2018 Select Papers by *Cells*’ Editorial Board Members”.

## 1. Assembly of the mTOR Signaling Complexes TORC1 and TORC2

The coordination of cell growth, cell size, organ shape and body plan is largely controlled by one serine/threonine kinase, the mechanistic target of Rapamycin (mTOR), which is, historically, also known as a mammalian target of rapamycin [1]. It has been described as an atypical protein kinase, because it is closely related to the phosphatidylinositol 3-kinase (PI3K) family of lipid kinases and represents the founding member of the small family of PI3K-related kinases (PIKK) [2]. Its name is based on the experimental approach that lead to the discovery of the two yeast proteins Tor1 and Tor2, which were found in a screen with the anti-fungal, bacterial macrolite rapamycin in *Saccharomyces cerevisiae* [3]. Rapamycin acts as an immunosuppressant in mammals and is used as prevention against the rejection of organ transplants. The name is derived from the fact that it was isolated for the first time from the bacterium *Streptomyces hygroscopicus* found on Easter Island (Rapa Nui) [4,5].

**Figure 1:**
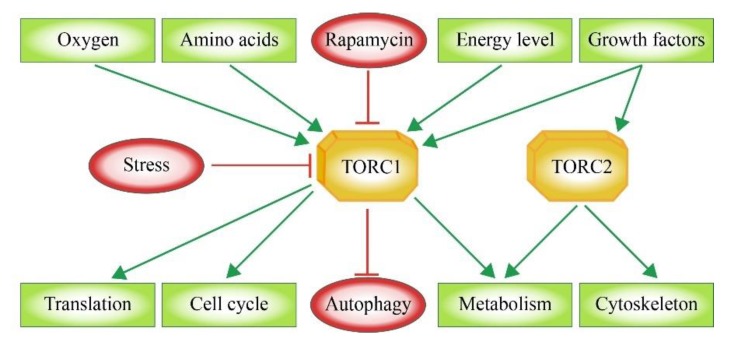
Cellular functions of TORC1 and TORC2. Growth factors activate both TORC1 and TORC2. Moreover, TORC1 integrates information concerning oxygen concentration, amino acid availability and changing energy levels, while it is inhibited by cellular stress and rapamycin. TORC1 supports translation, cell cycle, and cellular metabolism, while it inhibits autophagy. TORC2 controls cellular metabolism and cytoskeleton dynamics.

**Figure 2:**
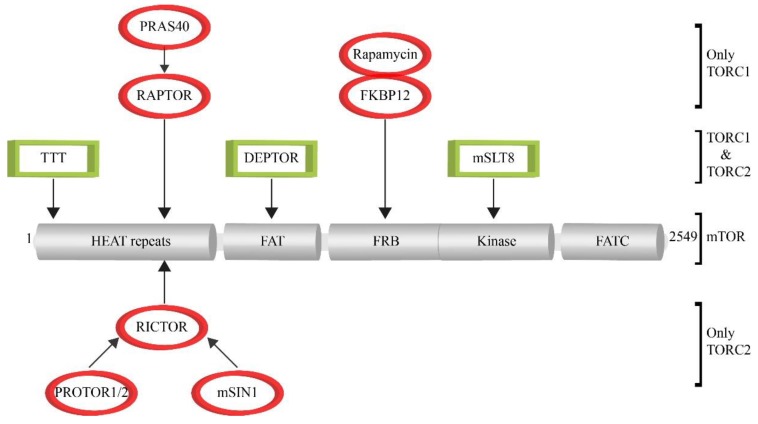
Composition of mTOR-complexes. The functional domains of the mTOR protein are depicted in the center. The binding factors present in both TORC1 and TORC2 are shown in boxes (TTT, DEPTOR, mLST8). The TORC1-specific factors are shown in ovals on top of the figure (RAPTOR, PRAS40, FKBP12-rapamycin), while the TORC2-specific factors are shown at the bottom (RICTOR, PROTOR1/2, mSIN1).

Mammals exhibit one mTOR protein, which represents the core component of two multi-subunit complexes. The TOR complex 1 (TORC1) integrates signals that sense the availability of amino acids, oxygen, growth factors as well as the cellular energy or stress levels (Figure 1). As a result, TORC1 promotes cell growth via its support of protein biosynthesis, cell cycle and cellular metabolism as well as the inhibition of autophagy. The TOR complex 2 (TORC2) functions mainly in the organization of the cytoskeleton [6,7] (Figure 1). The activity of TORC1 can be blocked by Rapamycin via an indirect mechanism. In this case, rapamycin forms an inhibitory complex by binding to the TOR-associated immunophilin FKBP12 (FK506 binding protein 12 kDa) [8,9]. TORC2 is rapamycin-insensitive. The binding site for Rapamycin-bound FKB12, the FRB domain (Figure 2), in mTOR is blocked by the TORC2-specific protein Avo3 in *S. cerevisiae* [10] and the mSIN1-RICTOR unit in mammals [11,12]. However, in mammalian systems it was shown that a long-term exposure to rapamycin abrogates mTORC2 signaling as a secondary effect. The rapamycin-associated mTOR may not be able to recycle from TORC1 in order to be incorporated into new TORC2 complexes [13,14].

The core complex of TORC1 consists of mTOR, RAPTOR (regulatory protein with mTOR) and mLST8 (mammalian lethal with Sec13 protein 8) [15,16,17] (Figure 2). RAPTOR binds to the HEAT repeat region in the amino-terminal half of mTOR. It functions as substrate adaptor, because it binds to the TOS (TOR signaling) motif that is present in several TORC1 substrates. Moreover, it is also involved in the correct lysosomal targeting of TORC1 [18,19]. Due to its important role, RAPTOR is the target of the endogenous negative-regulator protein PRAS40 (proline-rich AKT substrate of 40 kDa), which is together with DEPTOR (DEP domain containing mTOR interacting protein) [20,21,22] one of the two negative regulators of mTOR activity. mLST8 binds to the kinase domain of mTOR and is thought to support mTOR activity by stabilizing the kinase activation loop [23,24,25]. Therefore, it has been suggested that the mTOR and mLST8 hetero-dimer represents the core complex of TORC1 [26]. Several structural studies with mammalian and yeast TORC1 have addressed the composition of the complex as well as the inhibition mode used by Rapamycin to inhibit mTOR activity. Cryo-EM data have revealed that TORC1 forms a 1 mDa ‘lozenge’-shaped dimer. The contact sites that form the dimerization interface comprise the interaction between the HEAT domains of the two mTOR molecules as well as the association of mTOR of the first monomer-part with the RAPTOR of the second part [26,27,28]. The work with crystal structures has revealed that the FRB domain of mTOR is directly involved in the interaction with mTOR substrates. This kinase-substrate interaction is blocked when rapamycin-FKB12 binds to the FRB domain. Therefore, FRB functions as a gatekeeper, as FRB-bound rapamycin-FKBP12 inhibits mTOR activity by directly blocking substrate recruitment and restricting active-site access [24,29]. The TTT (TEL2-TTI1-TTI2)-complex functions as a chaperone for several PIKK family members and is also important as assembly factor and scaffold that stabilize TORC1 [30,31].

TORC2 also contains the constituents mTOR, mLST8, DEPTOR as well as the associated TTT-complex [30,32]. In contrast to TORC1, the adaptor protein RAPTOR is replaced by RICTOR (rapamycin insensitive companion of mTOR) as HEAT-domain binding module of TORC2 [20,33,34,35]. Moreover, RICTOR also binds to the regulatory factors PROTOR1/2 [36,37,38] and mSIN1 [39,40,41]. A recently published structures of the mammalian TORC2 reveal that mSIN1-RICTOR are located close to the FRB-domain of mTOR and, therefore, mediate the rapamycin-insensitivity of TORC2 [11,12].

The mTOR pathway is well conserved from yeast to man and, therefore, can be regarded as a major growth regulator in virtually all eukaryotic cells. *S. cerevisiae* has also two distinct mTOR-containing complexes (Table 1). In this case, TORC1 can contain either Tor1 or Tor2, while TORC2 contains Tor2 only. Moreover, homologs of RAPTOR (Kog1), mLST8 (Lst8), RICTOR (Avo3), as well as mSIN1 (Avo1) have been described for yeast. However, several additional complex constituents and regulating factors are specific for mammals or yeast (Table 1), based on the different requirements in the sensing of the vastly different environmental conditions that are relevant for unicellular and multicellular organisms [42,43].

## 2. Upstream Factors: Integration of Environmental Signals

**Figure 3:**
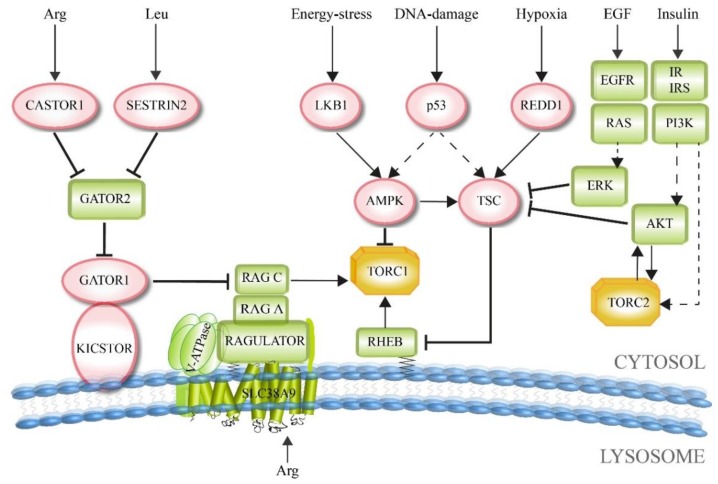
Upstream factors of the TOR pathway. The most important factors required for the signal integration of different stimuli by TORC1 and TORC2 are shown. Factors that enhance TOR activity are shown in squares, while those that hamper TOR activity are shown in circles. Dashed lines: effect mediated via additional proteins that are not depicted in the figure.

In general, TORC1 supports anabolism-linked reaction pathways. Therefore, the presence of enough energy equivalents and molecular building blocks within the cell stimulates TORC1 activity, which then promotes cellular growth. In contrast, inhibiting factors should signal the contrary information during fasting or cellular stress, namely the lack of enough resources required for further cell growth and proliferation, resulting in a block of TORC1 activity. This is the basic form that also more divergent and complex signaling TOR pathways are related to. Upstream signaling factors include growth indicators, energy level, oxygen status, DNA homeostasis, and amino acid concentrations (Figure 3).

Most growth factors that stimulate TORC1 are blocked by the tuberous sclerosis complex (TSC). TSC is a trimeric complex that comprises TSC1, TSC2 and TBC1D7 (TBC 1 domain family member 7) [44]. TSC2 functions as a GTPase activating protein (GAP), which is structurally stabilized and enzymatically stimulated by TSC1 and TBC1D7 [44,45,46]. TSC acts as GAP for the small GTPase RHEB (RAS homolog enriched in brain). On its own, the GTP-bound form of RHEB directly interacts with the catalytic domain of mTOR and, therefore, activates TORC1 [21,47,48,49]. In general, GTPases bind their interaction partners in the GTP-bound form. The stimulation of the GTPase activity by a GAP protein results in the hydrolysis of GTP and dissociation of the GTPase from the interacting partners, which ends the physiologic signaling output of the GTPase [50]. Here, TSC stimulates the GTPase activity of RHEB and, therefore, inactivates it, resulting in a downregulation of the RHEB-dependent support of TORC1 function.

On the other hand, TSC itself can be inactivated, when an upregulation of TORC1 signaling is again required. This relieve of the TSC-dependent inhibition of TORC1 is mediated via different growth factor pathways. The TSC2 subunit is phosphorylated and inactivated by the kinase AKT1 (Ak strain transforming 1), which was stimulated by the insulin/insulin-like growth factor-1 (IGF-1) pathway [51,52,53]. Moreover, AKT1 can also directly phosphorylate and inactivate the negative regulatory TORC1 subunit PRAS40 [21]. TSC2 can also be phosphorylated by the MAP kinase ERK (extracellular signal-regulated kinase), which had been activated via the receptor tyrosine kinase-dependent RAS pathway [54,55]. In summary, in many cases growth factors activate TORC1 by inhibiting the TORC1-negative regulator TSC.

In the context of the influence of the energy and oxygen status in TORC1 signaling, the central enzyme is the AMP-activated protein kinase (AMPK). It is activated during intracellular or environmental stress conditions that are caused by low ATP levels, DNA damage, or hypoxia. Therefore, AMPK acts as a metabolic regulator under stress conditions when cell growth is not favorable and should be limited.

The tumor suppressor LKB1 (liver kinase B1) is activated during energy stress, resulting in the phosphorylation and activation of AMPK [56]. Subsequently, AMPK can inhibit TORC1 indirectly by phosphorylating and activating TSC2 [57,58]. It also acts on TORC1 directly by phosphorylating and inactivating RAPTOR [59]. AMPK is indirectly activated during hypoxia, which is caused by the decreasing ATP-level [60]. Moreover, an additional layer of TORC1 inhibition is specifically mediated by the hypoxia-induced REDD1 (regulated in DNA damage and development 1), which binds and activates TSC [60,61].

The DNA damage-response is related to the cellular stress pathways. TORC1 is thought to be inhibited via the induction of p53 target genes, including the AMPK regulatory subunit (AMPKβ), PTEN and TSC2, resulting in an increase in TSC activity and therefore TORC1 inhibition [62].

TORC1 activation is tightly coupled to diet-induced changes in amino acid concentrations, because amino acids are not only essential building blocks of proteins, but also sources of energy and carbon for many other metabolic pathways [25]. Moreover, recent work from the fungus *Neurospora crassa* shows that a RAGULATOR-like protein is involved in amino acid sensing, as well as in the regulation of the circadian clock, strongly suggesting a coordinated link between circadian rhythms and TOR-dependent metabolism [63].

The RAG GTPase complex plays a central role in amino acid-based activation of TORC1 [64,65]. The RAG core complex consists of an obligate GTPase heterodimer, which can be formed by different combinations of either RAG A or RAG B with either RAG C or RAG D [66,67]. The RAG complex is bound to the lysosomal membrane through the pentameric RAGULATOR (LAMTOR) complex [68,69]. Upon stimulation with amino acids, the RAG proteins are converted into their GTP-bound state, which enables them to recruit TORC1 to the lysosome via an interaction with the RAPTOR subunit. This assembly also allows the interaction of TORC1 to lysosomal RHEB. This explains why both RHEB and RAG have to be activated in order to start TORC1 signaling and why both growth factors as well as amino acids have to be present for full activation of TORC1.

TORC1 senses cytosolic as well as intra-lysosomal amino acids via different mechanisms. The lysosomal amino acid transporter SLC38A9 functions as an arginine sensor for TORC1 [70,71,72,73]. SLC38A9 interacts with the v-ATPase associated RAGULATOR complex, which then functions as a guanine-nucleotide exchange factor (GEF) and therefore as activator of the RAG GTPase complex [68,74].

Cytosolic amino acids, as demonstrated for arginine and leucine, are sensed by TORC1 through the dynamic interplay of the GATOR1 and GATOR2 complexes [75]. The GATOR1 complex is tethered to the lysosomal membrane by the KICSTOR complex. GATOR1 functions as a GAP for the RAG A/B GTPases and, therefore, as an inhibitor of TORC1 [76,77]. In contrast, the GATOR2 complex acts as a positive regulator of TORC1. It does so by binding and inhibiting GATOR1 at the lysosome [75]. GATOR2 itself is blocked by CASTOR1 and SESTRIN2 under amino acid deprivation [78,79]. The association of arginine with CASTOR1 prevents the interaction of CASTOR1 with GATOR2. Similarly, leucine blocks the interaction of SESTRIN2 with GATOR2. Therefore, CASTOR1 functions as arginine sensor, while SESTRIN2 acts as leucine sensor in the context of TORC1 signaling [80,81,82,83,84]. Thus, both arginine and leucine stimulate TORC1 activity at least in part by releasing inhibitors from GATOR2, establishing GATOR2 as a central node in the sensing of amino acids by TORC1 [25]. However, there are also GATOR2-independent amino acid sensing modes. Glutamine is sensed via the RAG-related ARF family GTPases [85]. Another example is the FOLLICULIN-FNIP2 complex, which acts as a GAP for RAG C/D. RAG C/D is an unusual GTPase dimer, as it binds TORC1 not in its GTP-, but in its GDP-bound form [86]. Therefore, FOLLICULIN-FNIP2 acts as an amino acid-dependent positive regulator of TORC1 [86,87,88].

TORC2 is mainly regulated by the insulin/PI3K signaling pathway [25]. The presence of insulin and the downstream formation of the signaling lipid PtdIns(3,4,5)P3 (PIP3) activates TORC2. The TORC2 subunit mSIN1 exhibits the phosphoinositide-binding PH-domain, which is required for the insulin-dependent regulation of TORC2. The PH-domain of mSIN1 inhibits the catalytic activity of TORC2 in the absence of insulin [89]. Moreover, mSIN1 can be phosphorylated and activated by AKT1. This interdependence is an example for the existence of a positive-feedback loop. Partial activation of AKT1 promotes the activation of TORC2, which results in a phosphorylation of AKT1 by TORC2 [90].

Interestingly enough, TORC2 signaling can be inhibited by TORC1. GRB10, which is a negative regulator of insulin/IGF-1 receptor signaling upstream of AKT1, is phosphorylated and activated by TORC1 [91,92]. GRB10 disrupts the interaction of the insulin receptor with the downstream insulin receptor substrate 1 (IRS1). Moreover, IRS1 can be phosphorylated and inactivated by the TORC1 target S6K1, which results in a negative feedback loop of both TORC1 and TORC2 signaling [93].

## 3. Downstream Factors of mTOR Signaling

**Figure 4:**
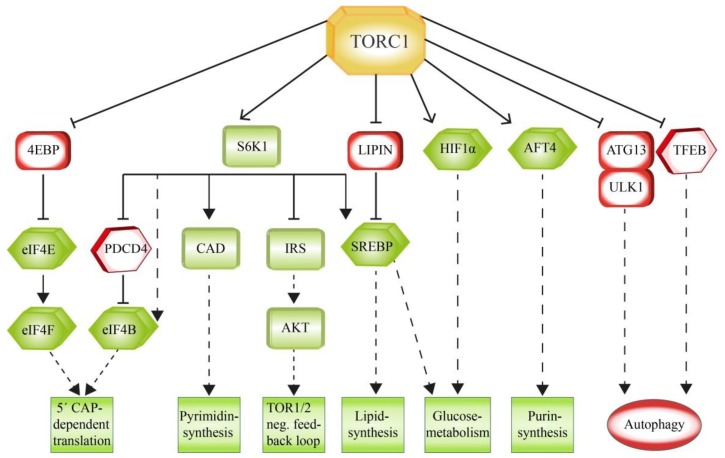
Downstream effectors of TORC1. The direct phosphorylation of targets by TORC1 has context-dependent results. Factors that interfere with the aims of TORC1 function are inactivated by phosphorylation, while supportive factors are stimulated by TORC1. Factors directly involved in transcription and translation are marked with hexagonal boxes.

TORC1 has a central role in controlling the balance of anabolism and catabolism in response to environmental conditions [32]. Since one of the main functions of TORC1 is to support cell growth, it also regulates the increased production of the required proteins, lipids and nucleotides. In line with this, it downregulates catabolic pathways like autophagy. In general, TORC1 regulates many of these processes via phosphorylation of translation-linked proteins (Figure 4).

Protein biosynthesis is promoted by TORC1 mainly through the direct phosphorylation of two key effectors, namely the ribosomal protein S6 kinase 1 (S6K1) and the eukaryotic translation Initiation factor 4B (eIF4E) binding protein (4EBP) [94].

S6K1 is a serine/threonine protein kinase that phosphorylates and activates eIF4B, which functions as a positive regulator of the 5’ cap binding eIF4F complex, as well as several other substrates that promote mRNA translation initiation [95]. Another S6K1 target is the eIF4B-inhibitor PDCD4. The phosphorylation by S6K1 results in the proteasomal degradation of PDCD4 [96]. While translation is supported by S6K1, it is inhibited by 4EBP, which binds the translation initiation factor eIF4E in order to prevent the assembly of the eIF4F complex. Like S6K1, 4EBP is also a direct target of TORC1. In this case the phosphorylation results in an inhibition of the target, as phosphorylated 4EBP dissociates from eIF4E and, therefore, allows the 5’ cap-dependent translation to occur [97,98]. Therefore, many pathways are regulated by TORC1 on translational level. While acute inhibition of TORC1 moderately downregulates mRNA translation in general, mRNAs containing the TOP (5’ terminal oligopyrimidine) or TOP-like motifs are affected in particular [99,100].

Since growing cells require sufficient amounts of lipids for the extension of their membranes, TORC1 promotes de novo lipid synthesis. TORC1 activates the sterol responsive element binding protein (SREBP) transcription factors, which control the expression of genes involved in lipogenesis [101]. Normally, low sterol levels activate SREBP. However, TORC1 signaling can also support SREBP independently of sterol levels via two distinct mechanisms: TORC1 activates S6K1, which then mobilizes SREBP [102], or alternatively, TORC1 phosphorylates and inactivates the SREBP-inhibitor LIPIN1 [103]. Growing and proliferating cells rely on an enhanced biosynthesis of nucleotides. TORC1 supports this process as well as the biogenesis of ribosomes [104,105]. Purine synthesis is supported by TORC1 via the phosphorylation of the activating transcription factor 4 (AFT4), which then mediates the expression of the enzyme methylenetetrahydrofolate dehydrogenase 2 (MTHFD2) as a central component of the mitochondrial tetrahydrofolate cycle that provides one-carbon units for this process [106]. Pyrimidine synthesis is promoted by TORC1 via S6K1, which phosphorylates and activates the carbamoyl-phosphate synthetase (CAD), a critical component of the de novo pyrimidine synthesis pathway [107,108].

Cellular growth is supported by TORC1 by the facilitation of the incorporation of nutrients into new biomass. This requires a TORC1-dependent shift in glucose metabolism from oxidative phosphorylation to glycolysis. TORC1 enhances the translation of the transcription factor HIF1a, which is involved in the expression of several glycolytic enzymes such as phosphofructokinase (PFK) [102]. Moreover, TORC1-dependent activation of SREBP results in an enhanced influx of glucose to the oxidative part of the pentose phosphate pathway. This pathway utilizes glucose in order to generate NADPH and, depending on the environmental fine tuning, either glycolysis intermediates or pentoses for nucleotide synthesis [109].

In contrast, TORC1 suppresses protein turnover mainly via the inhibition of autophagy. This is accomplished via posttranslational modification of autophagy key factors required for the formation of the autophagosome. ULK1 and ATG13 are early acting factors that are required for the initiation of autophagy and which are therefore phosphorylated and inhibited by TORC1 [110,111,112]. ULK1, for instance, is phosphorylated by TORC1 under conditions when enough nutrients are available. This inhibiting phosphorylation prevents the binding of ULK1 to and the activation by AMPK [111]. TORC1 inhibits autophagy also via transcription control. Under nutrient replete conditions, TORC1 phosphorylates the transcription factor EB (TFEB) and thereby blocks its nuclear translocation and therefore inhibits expression of genes for lysosomal and autophagosomal biogenesis [113,114,115].

The ubiquitin-proteasome system (UPS) is the other important pathway for protein turnover in the cell. Proteins that should be degraded are selectively marked by ubiquitination, which functions as recognition signal for the 26S proteasome. The different observations of the involvement do not fit into one coherent model yet. Acute TORC1 inhibition by rapamycin promotes proteolysis via ERK5 [116,117] in order to gain free amino acids for TOR1 re-activation, while prolonged TORC1 activation also seems to trigger an increased protein turnover via the transcription factor erythroid-derived 2-related factor 1 (NRF1) [118,119,120] in order to balance the increased rate of protein synthesis.

**Figure 5:**
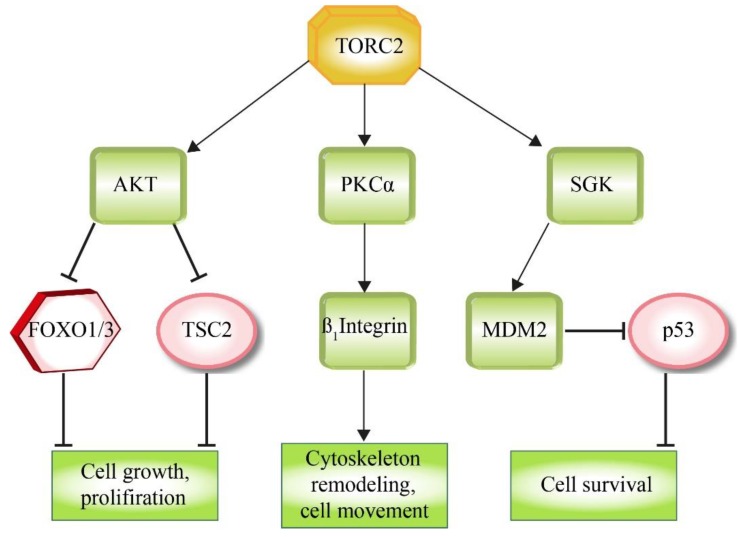
Downstream effectors of TORC2. The direct phosphorylation of the depicted kinases by TORC2 results in their activation. Factors that interfere with the aims of TORC2 (circles, hexagon) function are inactivated by phosphorylation, while supportive factors (squares) downstream of the TORC2 are stimulated by phosphorylation.

TORC2 controls cellular function and proliferation via the phosphorylation of several members of the AGC (PKA/PKG/PKC) family of protein kinases. Especially, members of the PKC subfamily, which regulate various aspects of cytoskeletal remodeling and cell migration, are TORC2 targets [33,34,121,122,123], like e.g., PKCδ, which has a central role in the upregulation of β_1_ integrin levels [124] (Figure 5). Another target of the AGC-kinase family is SGK (serum and glucocorticoid kinase), which regulates ion transport as well as cell survival, e.g., via the stabilization of the E3 ligase MDM2, which then blocks pro-apoptotic signaling by ubiquitinating p53 [125,126].

AKT1, which is a key effector of insulin/PI3K signaling [127], is phosphorylated and activated by TORC2 (Figure 3 and Figure 5). AKT1 supports cell survival, cell growth and proliferation via the phosphorylation and inactivation of several key factors, like the FOXO1/3a transcription factors or the TORC1 inhibitor TSC2 [40,128].

## 4. mTOR Activity Is a Metabolic Marker for the Potential Survival of Cancer Cells

Hyperactivation of TORC1 is linked to carcinogenesis via different mechanisms. Potential upstream factors are the tuberous sclerosis genes TSC1 and TSC2. Inherited mutations can cause autosomal-dominant hamartomas [129,130] and lymphangioleiomyomatosis [131]. Another important factor in this context is mutated and inactivated p53, which is in its active form a well described tumor-suppressor that downregulates cell division [132,133,134]. p53 acts against TORC1 by transactivating its negative regulators AMPK and TSC2 [62].

The downstream effects of mTOR in carcinogenesis are linked to the activation of a signaling network that enhances glycolysis via the upregulated expression of pyruvate kinase [135]. Pyruvate kinase (isoenzyme M2) is a well-described mediator of the so-called Warburg effect. The Warburg effect is a central metabolic characteristic of cancer cells that rely mostly on cytosolic substrate-level phosphorylation during anaerobic glycolysis and less on mitochondrial oxidative phosphorylation for ATP production [136,137]. Therefore, the survival of tumor cells under hypoxic conditions relies on a HIFa-dependent upregulation of glycolytic gene expression, resulting in energy production under nearly anaerobic glycolysis conditions [138,139]. Another important glycolytic enzyme is hexokinase isoform II (HK II), which is upregulated in several malignant tumors. The synthesis of HK II is stimulated by TORC1 via HIF1a in the presence of glucose and insulin, allowing the support of anaerobic glycolysis in cancer cells. However, under conditions when this pathway is hampered, e.g., under glucose deprivation or in insulin-resistant cells, HK II can bind directly to TORC1 and inhibit its function. As a result, the TORC1-mediated block of autophagy is relieved and the cells can use nutrients recycled from the lysosome, e.g., for gluconeogenesis [140,141].

The role of autophagy in health and disease has been described as a double-edged sword [142,143]. One the on hand, it enables tumor cell survival under stress conditions, while on the other hand it protects healthy cells against oncogenic transformation. Therefore, another downstream effect of TORC1 signaling in promoting carcinogenesis is based on its inhibition of autophagy [110,144]. In general, autophagy protects healthy cells by lowering the risk of genomic mutation via the removal of damaged ROS-producing organelles, like mitochondria and peroxisomes [145,146,147]. Recent meta-analyses strongly indicate that the defined and controlled inhibition of mTOR activity reduces the incidence of a variety of cancers [148,149].

In summary, TORC1 raises the possibility for the oncogenic transformation of cells via the inhibition of autophagy and it supports the survival of cancer cells via the promotion of glycolysis.

TORC2 activation has been shown to support tumor growth in combination with a loss of function of PTEN (phosphatase and tensin homolog), which antagonizes PI3K signaling [150]. Especially the support of lipogenesis by TORC2 has been shown to be associated with steatosis and certain kinds of cancers [151,152].

## 5. Involvement of mTOR Activity in Obesity and Diabetes

Enhanced activation of TORC1 and increased downstream signaling has been implicated in important metabolic diseases, such as obesity and diabetes. Prolonged activation of the mTOR signaling pathway in liver and skeletal muscle of obese rats suggested a possible role of mTOR in obesity-linked insulin resistance [153]. Moreover, TORC1 contributes to amino acid-induced insulin resistance via its direct target S6K1. Phosphorylated and activated S6K1 as well as phosphorylated and inactivated IRS1 can be detected in hyperaminoacidemia and postprandial hyperinsulinemia [154]. In principle, insulin activates TORC1 signaling via AKT1. After a threshold of TORC1 activity is reached, IRS1 is phosphorylated and inactivated, which finally downregulates insulin sensitivity in a negative feedback loop [155].

The activity of TORC1 has been shown to be required for the differentiation of adipocytes in mice and humans [156,157]. Interestingly, this effect was shown to be time- and context-dependent. While short-term inhibition of TORC1 by rapamycin causes TORC2-mediated insulin resistance [13,158], the long-term blockade of TORC1 was reported to reduce high-fat diet-induced obesity in mice [159,160].

These findings are important in the context of type 2 diabetes, which is characterized by insulin resistance in the expanding adipose tissue of obesity. The downregulation of the FOXO1 transcription factor, which is a TORC2 target, mimics the insulin-resistant state of type 2 diabetes in human primary adipocytes [161]. Moreover, TORC2 phosphorylates and stabilizes the ubiquitin-ligase FBW8 (F-box/WD repeat containing protein 8), which then marks IRS1 for degradation, resulting in insulin-resistance by prolonged TORC2 activation [93,162].

## 6. mTOR Signaling in Aging

The regulation of mTOR is a central factor during the aging process of diverse organisms. The reduction of mTOR signaling has been demonstrated to extend lifespan in *Caenorhabditis elegans* [163], *Drosophila melanogaster* [164,165], *Saccharomyces cerevisiae* [166,167], as well as *Mus musculus* [168]. Similarly, inhibition of proteins that are tightly associated with a positive signaling output of mTOR, like RAGULATOR of *C. elegans* or S6K1 of mice [169], also results in extended lifespan under the tested conditions. Therefore, mTOR is often regarded as the currently best studied target for pharmacological treatment to extend lifespan [170,171,172,173]. Interestingly enough, the other important factor that influences longevity is caloric restriction, which is defined as a reduction in nutrient intake without incurring malnutrition. Different tested caloric restriction conditions do not further extend lifespan in *S. cerevisiae*, *C. elegans*, or *D. melanogaster* when combined with a reduction in mTOR signaling, which strongly suggests that both share overlapping mechanisms [164,166,174]. The pivotal mediator of the TORC1-dependent nutrient-signaling network underlying longevity is the RNA polymerase III (POL III) [175], which is required for the synthesis of tRNAs needed for translation.

However, it seems the situation is more complex in mammals. Inhibition of mTOR was suggested to reduce S6K1-dependent transcription and general mRNA translation, resulting in a lower level of potential proteotoxic and oxidative stress on cellular level [169]. Moreover, downregulation of mTOR could be beneficial against aging by lifting the mTOR-dependent block of autophagy. This would allow a better clearance of protein aggregates or damaged organelles, which have been implicated in age-related processes [176,177]. Finally, it is assumed that the attenuation of adult stem cells plays a crucial role in aging, because the inhibition of mTOR pathways was shown to enhance the self-renewal capacity of both intestinal and hematopoietic stem cells in *M. musculus* [178,179], as well as germline stem cells in *D. melanogaster* [180].

The described observations have led to speculations that mTOR inhibition might extend lifespan and delay age-associated diseases also in humans. However, prolonged treatment of humans with Rapamycin leads to side effects such as immunosuppression and glucose intolerance. While the potential anti-aging effects are related to TORC1, the negative metabolic side effects are mainly due to the indirect inhibition of TORC2 after long time treatment. However, alternative dosing regiments of rapamycin, as well as the invention alternative drugs against TORC1 are under development [181].

## 7. mTOR and Age-Related Diseases

Promising results were obtained with mTOR inhibitors in the context of certain age-related diseases. Several therapeutic concepts for the treatment of neurodegenerative diseases are based on the upregulation of autophagy via the inhibition of TORC1 in order to induce the removal of harmful protein aggregates [182,183]. Pharmacological inhibition of TORC1 via Rapamycin or the chemically-synthesized analogue Temsirolimus (CCI-779) were demonstrated to upregulate autophagy in model cells for neurodegeneration [182]. A corresponding decrease in cytotoxicity was detected in mouse, zebrafish, and *Drosophila* studies [184]. Publications about neurodegenerative diseases like Alzheimer`s disease (AD) suggest a promising treatment via the inhibition of TORC1 by rapamycin or rapamycin-analogues like Temsirolimus, resulting in a stimulated autophagy [185,186,187].

For the treatment of Parkinson`s disease (PD), several natural compounds, like curcumin derived from the curry spice turmeric, were shown to downregulate mTOR signaling. As a consequence, the elevated activity of autophagy cleared α-synuclein in animal and human cell models [188]. Curcumin is supposed to activate the protein phosphatase 2 and a calyculin A-sensitive phosphatase, which target and inhibit AKT, mTOR, and certain downstream factors like 4E-BP1 [188,189,190].

Inhibitors of mTOR are also used for the treatment of cardiovascular diseases. The functional role of mTOR in the cardiovascular system is context dependent. The mTOR complexes have been described as essential regulators of cardiovascular embryonic development and are also required for the postnatal preservation of cardiac structure and its adaptation to stress. This includes the cardiac adaptation to mechanical stress, which limits cardiomyocyte death and contributes to the development of compensatory hypertrophy. However, TORC1 activity in the heart during chronic stress has also been shown to have multiple maladaptive effects, which results in the pathological hypertrophy [191]. The treatment of cardiac hypertrophy can be supported by mTOR inhibition. Rapamycin was shown to reduce cardiac hypertrophy and improve cardiac function in mice [192,193]. Moreover, inhibition of mTOR signaling via AKT during eccentric hypertrophy was blocked by additional use of AKT inhibitors [194].

Another cardiovascular topic linked to mTOR function is atherosclerosis. In order to reduce the risk of atherosclerosis, inhibition of mTOR induces autophagy and depletes plaque macrophages. However, because the roles of mTOR in lipogenesis and insulin signaling are also blocked, common side effects of their use are dyslipidemia and insulin resistance, which are both risk factors for atherosclerosis. In order to minimize these effects, which would lead to an increase of low-density lipoprotein cholesterol levels, additional use of cholesterol lowering drugs is a recommended strategy [195,196,197].

## 8. Pharmacological Use of Different mTOR Inhibitor Families

After the discovery of Rapamycin, this macrolide compound was used as an antifungal drug against infections with *Aspergillus fumigatus*, *Candida albicans* and *Cryptococcus neoformans* [198]. When the important functional role of mTOR became evident during the basic research work with rapamycin, the combination of rapamycin with cyclosporine A was established as an important immunosuppressant against transplant rejection because of the inhibition of T-cell proliferation [199]. Moreover, based on its cytostatic activity, rapamycin could be used as an anti-cancer agent. More recently, rapamycin has also shown to contribute to the prevention of coronary artery restenosis [200] as well as to the treatment of neurodegenerative diseases [201]. New inhibitors of mTOR are being designed.

The so called first generation of mTOR inhibitors (Table 2) comprises the natural compound rapamycin (generic name: Sirolimus) and its engineered derivates, the so called rapalogs [202]. They have in common that they also bind to FKBP12, but they are supposed to have context-dependent and more favorable pharmacokinetic profile when compared to rapamycin.

Temsirolimus (Torisel) is the prodrug of rapamycin and is often used against renal cell carcinoma [149]. Everolimus is a rapalog that is used in transplantation medicine under the names Zortress or Certican, as well as in oncology for general tumor under the names Afinitor or Biocon.

The second generation of inhibitors targets both TORC1 as well as TORC2 by competing with ATP at the catalytic site of the mTOR kinase, which is present in both complexes [202,203]. Similar to rapalogs, they can decrease protein translation and attenuate cell proliferation in several cancer cell lines [204,205]. Along with the directly kinase-dependent functions of mTOR, the second generation inhibitors also block the feedback activation of the PI3K and AKT signaling pathways. Therefore, in addition to the optimized inhibition of TORC1 in rapamycin-resistant cell lines, these inhibitors are thought to block TORC2 as well as interfere with the interplay with the PI3K and AKT [206,207]. Another approach is to target mTOR associated proteins, like the inhibition of RHEB by the small molecule NR1, which inhibits TORC1-dependent phosphorylation of S6K1 [208].

The third generation of mTOR inhibitors is supposed to be used in cells that have developed a resistance against both first- and second generation inhibitors [209]. Theses inhibitors are bivalent molecules that exploit the juxtaposition of the corresponding two drug-binding pockets. RapaLink-1 consists of a rapamycin-FRB compound linked to the mTOR kinase inhibitor TORKi [210,211]. Therefore, exploitation of both the kinase domain as well as FRB domain of mTOR should potentially inhibit mTOR-related dysfunctions in the context of tumor growth [210,212]. Moreover, the methodological approach to design novel bivalent inhibitors could be applied to resistances in other disease-relevant signaling pathways.

## 9. Conclusions

The PIKK-type kinase mTOR plays a central role as coordinator of cellular metabolism by integrating distinct extracellular stimuli and intracellular signals for the initiation of a concerted and adjusted response. Because of its crucial role in normal physiology, the dysregulation of mTOR signaling is often associated with certain diseases as well as the molecular process of aging. For example, its function in glucose and lipid metabolism links it to the occurrence of obesity and diabetes, while its function in promoting cell proliferation and inhibiting autophagy often associates it with tumor formation. Therefore, several key factor in the downregulation of mTOR signaling, like the TSC complex, have been described as tumor suppressors. A better understanding of the different TORC1 and TORC2 signaling pathways will be of importance for the further development of drugs that modulate mTOR functions. This could involve two basic strategies. The first concerns the identification and characterization of further cross-talk between different mTOR-dependent pathways and their effectors. Moreover, it will be beneficial to be able to discriminate different pathway-selective domains in mTOR itself or in individual complex components that could be targeted selectively by then pathway-specific drugs. With this information as a prerequisite, the second approach based on clinically-oriented research will pursue the search for the synergistic combination of distinct drugs that might modulate mTOR signaling by acting both on mTOR itself, as well as on individual TORC1 or TORC2 effector proteins in addition. Moreover, it has to be taken into account that the drug-dependent modulation of mTOR activity has to differ between acute and chronic disorders. Therefore, the identification of candidate inhibitors with novel mechanisms of action as well as the definition of prognostic and predictive biomarkers associated with different mTOR activity levels will enable the chance on a new generation of effective and personalized disease treatment in the context of a “bench-to-bedside” approach.

## Figures and Tables

**Table 1:** Function and evolutionary conservation of TOR complex constituents. The table lists the constituents of TORC1 and TORC2 in different species and describes their molecular function. Common factors are in green, while unique factors are in red.

	*H. sapiens*	*S. cerevisiae*	*D. melanogaster*	*C. elegans*	Function
TORC1	mTOR	Tor1, Tor2	TOR1	TOR	Serine/threonine kinase
	TEL2, TTI1, TTI2	Tel2, Tti1, Tti2	Tel2, Tti1, Tti2	clk-2	Assembly and stability
	mLST8	Lst8	Lst8	lst-8	Core complex
	DEPTOR	-	-	-	mTOR inhibitor
	RAPTOR	Kog1	Raptor	daf-15	Localization; substrate binding
	FKBP12	Fpr1	FKBP12	fkbp-2	PPlase; binds rapamycin
	PRAS40	-	-	-	mTOR inhibitor
	-	Tco89	-	-	unknown
TORC2	mTOR	Tor2	TOR	TOR	Serine/threonine kinase
	TEL2, TTI1, TTI2	Tel2, Tti1, Tti2	Tel2, Tti1, Tti2	clk-2	Assembly and stability
	mLST8	Lst8	Lst8	lst-8	Activator of TORC2 kinase activity
	DEPTOR	-	-	-	mTOR inhibitor
	RICTOR	Avo3	Rictor	rict-15	Interaction with substrates; blocks FKBP12/Rapa
	PROTOR	Bit61, Bit2	-	-	Increase of SGK1 activation
	mSIN1	Avo1	Sin1	sinh-1	Interaction with SGK1
	-	Avo2	-	-	Cytoskeleton regulation

**Table 2:** Pharmacological use of different mTOR inhibitor classes. The table displays the characteristics of the corresponding inhibitor family and lists important examples.

**First Generation**
Target: TORC1 (binding to FKBP1)
Examples: Sirolimus (prophylaxis organ rejection) Temsirolimus (renal cell carcinoma) Everolimus (renal cell carcinoma) Status: FDA/EMEA approved for certain applications
**Second Generation**
Targets: TORC1,TORC2,PI3K (bind kinase domain) Examples: NVP-BEZ235 (PI3K/TORC1/TORC2) PF-04691502 (PI3K/mTOR) OSI-027 (TORC1/TORC2) Status: Different preclinical and clinical stages
**Third Generation**
Targets: TORC1,TORC2 (bivalent binding to FRB domain and kinase domain) Example: RapaLink-1 (rapamycin-FRB-binding compound linked to TORKi) Status: Preclinical research
